# A feasibility study of mHealth and wearable technology in late onset GM2 gangliosidosis (Tay-Sachs and Sandhoff Disease)

**DOI:** 10.1186/s13023-020-01473-x

**Published:** 2020-08-03

**Authors:** Elin Haf Davies, Jean Johnston, Camilo Toro, Cynthia J. Tifft

**Affiliations:** 1 Aparito Limited, Unit 11 Gwenfro, Wrexham Technology Park, Wrexham, Wales LL13 7YP UK; 2 Office of the Clinical Director, National Human Genome Research Institute, National Institutes of Health, Bethesda, MD USA

**Keywords:** Late onset GM2 gangliosidosis, Wearable technology, Mobile health, lysosomal storage disease

## Abstract

**Background:**

As part of a late onset GM2 gangliosidosis natural history study, digital health technology was utilized to monitor a group of patients remotely between hospital visits. This approach was explored as a means of capturing continuous data and moving away from focusing only on episodic data captured in traditional study designs. A strong emphasis was placed on real-time capture of symptoms and mobile Patient Reported Outcomes (mPROs) to identify the disease impact important to the patients themselves; an impact that may not always correlate with the measured clinical outcomes assessed during patient visits. This was supported by passive, continuous data capture from a wearable device.

**Results:**

Adherence rate for wearing the device and completing the mPROs was 84 and 91%, respectively, resulting in a rich multidimensional dataset. As expected for a six-month proof-of-concept study in a disease that progresses slowly, statistically significant changes were not expected or observed in the clinical, mPROs, or wearable device data.

**Conclusions:**

The study demonstrated that patients were very enthusiastic and motivated to engage with the technology as demonstrated by excellent compliance. The combination of mPROs and wearables generates feature-rich datasets that could be a useful and feasible way to capture remote, real-time insight into disease burden.

## Introduction

The GM2 gangliosidoses, Tay-Sachs (TSD) and Sandhoff (SD) diseases, are neurodegenerative disorders, caused by a deficiency of the lysosomal enzyme beta- hexosaminidase A (Hex A). The deficiency causes accumulation of GM2 ganglioside particularly in neurons where the rate of ganglioside synthesis is the highest, leading to progressive neurodegeneration. Although the incidence of TSD and SD is very low (1 in 320,000 for TSD and even less frequent for SD [1]) there are common mutations in ethnic populations that make it more frequent. In the Ashkenazi Jewish population, the disease incidence of infantile TSD is about 1 in every 3500 newborns. Similarly, there is a common mutation (*HEXA*, p.GLY269SER) in the eastern European population that accounts for many of the individuals with late onset TSD [2]. In contrast to infantile TSD or SD disease the late-onset forms have symptom onset in adolescence or early adulthood, with ataxia, selective and progressive muscular atrophy leading to increased falls and difficulty rising from a chair or the floor, and for TSD patients, dysarthria. The heterogeneity of the disease may also result in the misdiagnosing of older adults who have the disease, and a history of neuronal symptoms, through conflation with the clinical indications of other neurodegenerative disorders [3]. SD patients may often have tingling, numbness or pain in their hands and feet as a presenting sign.

There is currently no cure for TSD or SD. Research is focused on increasing HexA activity by enzyme replacement therapy where the blood brain barrier has been a formidable obstacle; by substrate reduction of ganglioside precursors using small molecules; or by gene delivery [4, 5]. As new treatment options emerge, it is imperative to identify and validate appropriate outcome measures by which to evaluate potential therapeutic effects.

We believe that these measures should include patient-reported outcomes, to provide the patient’s perspective and give them a voice in their own health care [6]. The development of smartphone applications has made it possible to collect this information easily and often [7]. In addition, wearable devices can continuously measure the quality and quantity of physical activity [8, 9], providing valuable information on motor function.

The aim of this study was to assess the feasibility of using digital health technology to monitor GM2 patients remotely between hospital visits. The technology included a wearable device and a smartphone application to record patient-reported outcomes. This proof-of-concept study also focused on capturing patient feedback on use of the technology and exploring the outcome data it can provide. We plan to extend use of the technology to validate outcome measures that monitor disease progression, measure the effects of therapeutic intervention, and solicit further patient feedback on the impact of the disease on their activities of daily living.

## Results

Eight consenting patients took part in the study and remained engaged for its duration. Age ranged from 28 to 61 years (44 ± 11), with three men and five women.

Laboratory and clinical results measured by clinical evaluation over the 6-month course of the study can be seen in Table 1. There were no statistically significant differences between baseline and month six in any of the measures.

**Table 1:** Laboratory and clinical data at baseline and six months (mean ± standard deviation) and statistical significance results

	Baseline	Month 6	***Wilcoxon p-value***
6MWT (meters)	316.88 ± 123.26	345.50 ± 117.68	0.11
BARS score	9.75 ± 6.09	10.06 ± 6.96	0.59
Neuroglyphics Off Target-dominant (%)	19.00 ± 14.11	16.62 ± 10.91	0.69
Neuroglyphics Off Target- Nd (%)	21.19 ± 13.99	17.77 ± 11.65	0.47
BARS Upper score	2.63 ± 2.22	2.75 ± 2.84	0.79
9HP Dom Avg (sec)	28.83 ± 8.23	27.22 ± 7.84	0.38
9HP Dom z-score (sec)	4.35 ± 2.77	4.04 ± 2.77	0.38
9HP Nd Avg (sec)	30.89 ± 15.88	29.84 ± 11.41	0.78
9HP Nd z-score (sec)	4.71 ± 5.39	5.05 ± 4.96	1
GAITRite data:			
Cadence (steps/min)	94.33 ± 18.86	99.95 ± 13.88	0.15
Velocity (cm/sec)	97.86 ± 34.08	108.59 ± 33.61	0.15
Step Length (cm)	60.10 ± 12.74	63.68 ± 14.62	0.38
Step Width (cm)	11.72 ± 3.77	11.53 ± 3.46	0.55
Step Time (sec)	0.67 ± 0.15	0.61 ± 0.09	0.11

*Avg* Average, *BARS* Brief Ataxia Rating Scale, *cm* Centimetres, *DOM* Dominate *min* Minute, *6MWT* 6-min walk test, *Nd* Non-dominant, *sec* Seconds, *9HP* 9-hole peg test – a brief, standardized and quantitative test of upper extremity (hand and arm) function z-score: calculated by converting raw into a common metric

### Adherence

Adherence to wearing the device ranged from 35 to 96% in terms of each individual patient over the 6-month period of the study. The median cohort adherence rate was 84%. Wearable usage decreased slightly from 3 months to 6 months primarily due to decreased usage over a holiday period and the coinciding battery life limits. The mean (standard deviation) number of daily steps for the cohort of eight patients was 7253.2 (490.0) with a median of 6526.9 steps. Complete data are seen in Table 2.

**Table 2:** Individual Patient Wearable Data and adherence rates (complete data)

Patient Number	001	002	003	004	005	006	007	008
**Wearable data**								
**Total Number of Days**	186	186	186	186	185	185	185	185
**Total Number of Days Active on Wearable**	157	179	65	157	164	122	121	164
**Average Daily Steps**	10,147.7 ± 1509	6432.8 ± 290.79	4122.1 ± 318.8	4560.6 ± 208.21	12,424.6 ± 323.13	9342.7 ± 178.8	5909.6 ± 689.3	4041.7 ± 196.6
**Adherence rate (%)**	84	96	35	84	89	66	65	89
**Patient Reported outcomes (PROs)**								
**Adherence rate (%)**	97	95	91	82	91	75	63	96

*SD* standard deviation

**Fig. 1:**
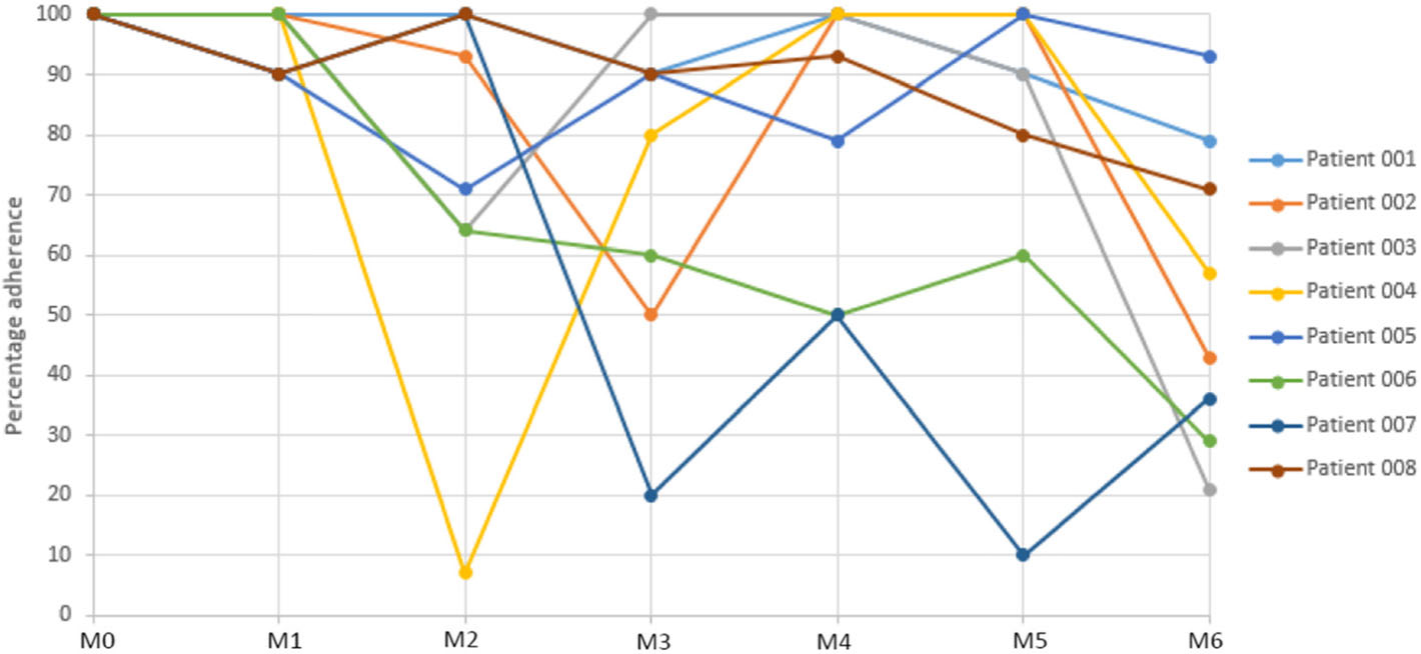
Individual Patient Adherence to Completion of PRO Data

For the wearable data, the median adherence rate i.e. calculated when the patient completed a minimum of 8 × 30-min epochs of data, was 91% (range: 63–97%). All patients gave at least two responses to each PRO over the 6-month period, but adherence to the PROs was variable by patient and month and overall tended to decrease towards the end of the study (see Fig. 1).

### Wearable data

**Fig. 2:**
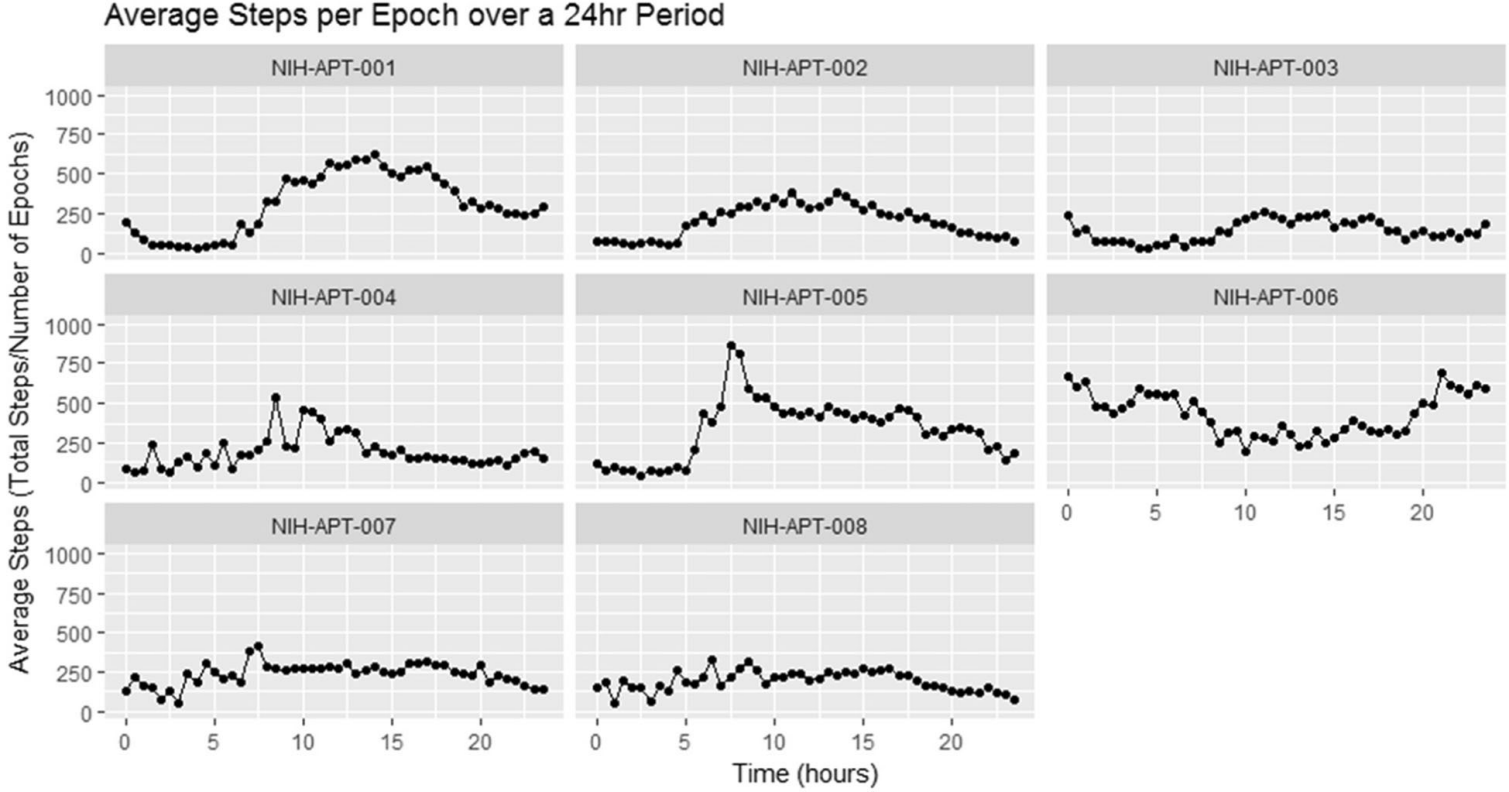
Average number of steps per 30-min epoch over a 24-h period

The average steps per epoch over a 24 h Period (from midnight to midnight) is illustrated in Fig. 2. On average, less activity was recorded between midnight and 7 am, consistent with average sleep patterns. Patient NIH-APT-006 who reported activity above 250 average steps per epoch at night worked night shifts.

Three wearable metrics were calculated (described in more detail in the Methods section): the average daily maximum (ADM), average daily steps (ADS), and average daily steps per 30-min epoch (ADE). Cohort analysis of ADM, ADS, ADE is presented in Table 3. No statistically significant changes were observed between baseline and month six.

**Table 3:** Cohort averages and statistical significance results for the wearable metrics

Mean (Median)	M0-M1	M1-M2	M2-M3	M3-M4	M4-M5	M5-M6	Overall Cohort Mean (Median) [range]	***Wilcoxon p-value***
**ADM**	1171.6 (1063.2)	1183.2 (954.1)	952.0 (893.7)	1094.1 (935.9)	1011.3 (1065.6)	943.5 (811.1)	1059.6 (962.3) [684 to 1625]	0.10
**ADS**	6902.7 (6452.9)	6850.6 (6867.9)	7825.7 (7068.2)	8431.5 (6525.0)	7317.2 (5955.8)	6346.6 (4971.1)	7253.2 (6526.9) [2532 to 16,315]	0.51
**ADE**	288.9 (240.8)	264.2 (208.3)	272.8 (236.0)	277.8 (238.4)	260.0 (281.8)	259.5 (246.3)	270.6 (235.8) [125 to 475]	0.80

*ADM* Average daily maximum, *ADS* Average daily steps, *ADE* Average daily steps per 30-min epoch. *M* month

### Clinical event data

Every single patient used the app to record their symptoms (range: 8–79 events reported). In terms of the number of patients who reported each event respectively, seven patients reported a fall/near fall (66 events), and six patients reported choking/coughing (67 events). Other symptoms reported were Tremor (10 events), Other (49 events), and Other Illness (72 events).

Other Illness, which covered a broad range of options (“Vomiting”, “Headache”, “Cold”, “Cough” and “Diarrhea”), was the most frequently reported event from the pre-selected options (72 events), while missed college/ work was the least reported event (5 events).

Five patients reported ‘Other’ events using free text. Of those, health-related responses were hiccups, leg/hip muscle spasm, headache, injuring arm, having an appointment with a physician because of feeling tired, lower back pain, acid reflux, short term memory, fall, migraine, neuropathy to right hip, muscle cramp, incontinence, sharp pain to body parts, numbness/tingling, bone grinding such as in the hip and taking medication such as Ibuprofen and Tylenol.

These self-reported clinical events are of paramount importance not only on their own but also to put context around the objective data of the wearable. In addition, the ability to report in real-time reduces the impact of memory recall on the details provided.

### mPRO data

Table 4 shows the Rosenberg Self Esteem scale, which is a widely used and validated self-esteem measure with a scale of 0 to 30, with a score less than 15 indicating potential problematic low self-esteem. Our cohort average ranged from 14.4 to 15.4 suggesting that this cohort are on the low side of self-esteem [10]. PedsQL fatigue scores ranged from 46.5 to 61.1 on a scale of 0 to 100, indicating fatigue in this cohort [11]. Self-reported “Impact on Family” was scored higher than “Impact of Disease”.

**Table 4:** mPRO and QoL scale results across the 6-month period with overall cohort averages and statistical significance

Mean (Median) [min-max]	M0	M1	M2	M3	M4	M5	M6	Overall Cohort	***Wilcoxon p-value***	***Hollander p-value***	Reference range
Tremor impact	0.0 (0.0) [0.00–0.00]	0.4 (0.0) [0.00–1.00]	0.8 (0.5) [0.00–2.00]	1.0 (1.0) [0.00–2.00]	0.7 (0.5) [0.00–2.00]	0.9 (1.0) [0.00–2.00]	1.3 (1.0) [0.00–2.00]	0.7 (0.5) [0.00–2.00]	0.05	0.9375	0–3 Higher score: more tremor
Disease Impact	4.5 (5.0) [2.00–7.00]	4.9 (4.5) [0.00–10.00]	4.1 (4.0) [1.00–6.00]	5.8 (6.5) [1.00–10.00]	5.6 (5.0) [2.00–9.00]	6.1 (5.0) [3.00–10.00]	5.7 (5.5) [3.00–9.00]	5.2 (5.0) [0.00–10.00]	0.20	0	0–19 Higher score = higher impact
Impact on the family	6 (6.0) [2.00–9.00]	6.1 (6.0) [4.00–9.00]	5.6 (6.0) [2.00–8.00]	6.3 (6.0) [5.00–8.00]	6.3 (6.0) [5.00–8.00]	6.1 (6.0) [5.00–8.00]	6.3 (6.5) [5.00–8.00]	6.1 (6.0) [2.00–9.00]	0.79	0	0–12 Higher score = higher impact
Wider impact	1.3 (0.0) [0.00–7.00]	1.3 (0.0) [0.00–7.00]	2.6 (1.0) [0.00–7.00]	1.2 (0.0) [0.00–6.00]	1.6 (0.0) [0.00–7.00]	0.9 (0.0) [0.00–5.00]	1.8 (1.0) [0.00–7.00]	1.5 (0.0) [0.00–7.00]	1.0	0	0–14 Higher score = higher impact
Impact Composite Scale	0.27 (0.28) [0.11–0.40]	0.28 (0.31) [0.10–0.40]	0.29 (0.27) [0.08–0.44]	0.32 (0.34) [0.15–0.45]	0.27 (0.25) [0.00–0.49]	0.30 (0.29) [0.21–0.40]	0.31 (0.31) [0.23–0.40]	0.27 (0.29) [0.00–0.49]	0.42	0	0–1 Higher score = higher impact
Perceived Stress	17.9 (18.5) [11.00–26.00]	NA	19.6 (21.0) [7.00–25.00]	NA	16.3 (18.0) [9.00–24.00]	NA	20.0 (20) [12.00–29.00]	18.4 (19.3) [7.00–29.00]	0.04	0.8742	0–40 Higher score = higher stress
Global Self Worth	17.0 (17.5) [12.00–24.00]	NA	16.0 (15.5) [13.00–22.00]	NA	17.8 (18.0) [14.00–22.00]	NA	18.1 (18.0) [14.00–24.00]	17.2 (17.8) [12.00–24.00]	0.59	0.5	0–24 Higher score = higher self-worth
Rosenberg Self Esteem	15.0 (15.0) [11.00–17.00]	NA	15.3 (15.5) [11.00–19.00]	NA	14.4 (14.0) [13.00–16.00]	NA	15.4 (15.0) [13.00–19.00]	15.0 (15.0) [11.00–19.00]	0.68	0	0–30 Higher score = higher self esteem
CHU 9D	0.87 (0.88) [0.67–0.96]	NA	0.84 (0.83) [0.66–1.00]	NA	0.83 (0.83) [0.68–0.92]	NA	0.83 (0.84) [0.72–0.95]	0.84 (0.83) [0.66–1.00]	0.36	0.438	0.33–1 Higher score = better health
PedsQL Multi-dimensional Fatigue	49.5 (46.5) [31.94–73.61]	46.5 (43.1) [27.78–75.00]	46.7 (43.1) [38.89–66.67]	49.7 (45.8) [34.72–73.61]	46.6 (45.1) [29.17–75.00]	50.2 (50.0) [31.94–83.33]	61.1 (58.3) [50.00–75.00]	49.6 (45.8) [27.78–83.33]	0.73	0	0–100 Higher score = less fatigue

*CHU 9D* Child Health Utility 9D, *M* month

### Health care visits data

Seven out of eight patients used the app to report healthcare visits at least once. Number of Visits responses ranged for each individual patient from 0 to 65 for Healthcare Professional (*n* = 117), 0–2 for General Practitioner (*n* = 4), and 0–3 for Hospital (*n* = 3). Healthcare Professional was the most reported healthcare visit at 117.

### Correlations

**Fig. 3:**
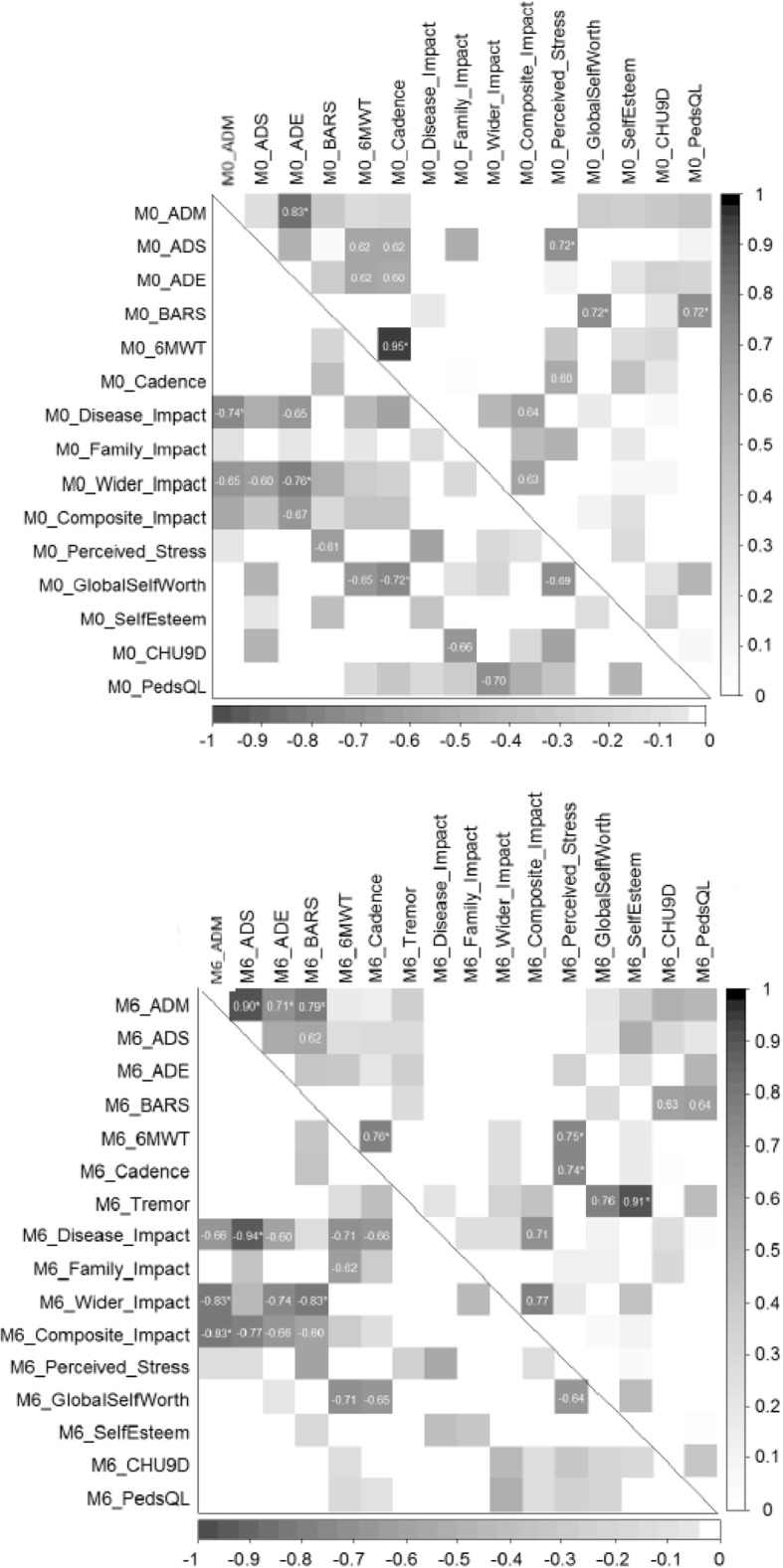
Correlations between clinical measures, wearable and mPRO data for baseline (top) and month 6 (bottom). The upper triangles show the positive correlations, and the lower triangles show the negative correlations: the darker the colour, the higher the correlation. Note that the Tremor scale is not included in the baseline correlations, as it was zero for all patients

Correlations were calculated between the three-wearable metrics (ADM, ADS, ADE), three clinical measures (6-min walk test (6MWT), Brief Ataxia Rating Scale (BARS), and cadence from the GAITRite walking assessment) and the ten mPROs at baseline and at month-6. For clarity, Fig. 3 highlights only results with moderate to strong correlations (coefficients > 0.6 or < − 0.6), and *p* < 0.05 is indicated by an asterisk.

Some of the wearable metrics are correlated with each other at month 0, with the highest positive correlations between the clinical walking assessments (6MWT and GAITRite cadence; 0.96), and between ADM and ADE (0.83) and the highest negative correlation seen between disease impact (i.e. impact of late onset GM2) and ADS (0.94) which may suggest that higher physical activity measured with the wearable device is linked to better walking performance and lower disease impact. The clinical walking assessments are also negatively correlated with the Impact Scales, but to a lesser extent than the wearable metrics. BARS score does not show any correlation with 6MWT or GAITRite. There were stronger correlations seen at month 6 when compared to month 0 between the three wearable metrics and impact factors. Stronger correlations were seen at month 6 than month zero between the three wearable metrics and impact factors.

### Feedback survey

A feedback survey was conducted at the end of the study and indicated that all eight patients considered the app to be “valuable” for reporting their symptoms to their doctor in real time, with four patients stating the app to be “very valuable”. Overall, 37.5% of patients said they were definitely likely to continue wearing the wristwatch and use the phone app on a long-term basis. A “very good” overall impression was reported by two out of eight patients, one reported their overall impression as “good”, and four as “ok”. This feedback was instrumental in the redevelopment of the app and the introduction of a new wearable.

## Discussion

This feasibility study demonstrated that utilizing mHealth with wearable technology was well accepted by patients over a six-month natural history study. Adherence to wearing the device remained greater than 65% throughout the six-month period for seven out of eight patients.

Engagement with the app (symptoms and mPROs) was utilized by all patients over the course of the study. In fact, at the end of the study some of the patients chose to continue to use the technology. It was noted that patient 003 had low adherence with respect to the wearable data but high adherence to the PROs. An explanation for this is that this patient experienced issues with the band on her device, which broke. A spare device was also sent to this patient which resulted in data loss.

Engagement with the app for Events indicates its value in patients monitoring their symptoms in real-time. Collecting patient-generated data outside of the hospital setting, for example, during drug development, enables healthcare professionals to capture data remotely on a real-time basis. This not only enables researchers and healthcare professionals to capture disease changes, but also reduces the burden on the healthcare system because fewer hospital-based assessments may be needed, either during a clinical study or for clinical practice. This also means patients benefit from having to attend fewer hospital appointments. The additional value of machine learning /artificial intelligence (ML/AI) provides additional support for the clinical value of the device/app, which can’t be implemented by human resources.

The clinical data (Table 1) suggest that the physical ability of the patients in the 6MWT remained the same or slightly improved over the six months. Likewise, all the GAITRite parameters tended to be higher at month six, but the increase was not statistically significant. The BARS assessment remained stable over the duration of the six-month study. This indicates that disease state as measured by these parameters remained stable during this relatively short observation period for a disorder with a documented slow progression.

Figure 2 shows the average steps per epoch over a 24 h period. Measuring such repeated patterns in longitudinal data collection can identify patterns and routines specific to each patient. Specific patterns that arise from commute and work breaks could be identified, and act as indicators of disease progression when things change. Patients with very low-level levels of activity in a month, i.e. engagement with wearing the device, had their data for any month excluded from analysis if the number of active days in that month was less than six days. It should be noted that the specific wearable device used was not able to differentiate between data captured while being worn by the patient or not, so patients with low activity might have had their activity discounted if they had not been active for a total of 8 × 30-min epochs.

The decrease in wearable usage seen during the period from 3 to 6 months is thought to be largely because of decreased usage over the holiday period and the coinciding battery life limits. Several of the patients needed to replace the batteries in the wearable device, therefore losing a few days of data.

Table 3 shows that there were no changes in the wearable metrics (defined as ADM, ADS, and ADE), in the six-month period of the study. This is consistent with the hospital-based assessment of 6MWT and GAITRite. As the study started in August and finished in February, the mild decrease noted could be linked to seasonal variation and changes in the weather. The ADS values obtained from patients were are high. This in part may have been as a result of the patients being conscious of the wearable monitoring their ambulatory activity, thus increasing their motivation. Prior studies have shown the use of pedometers to increase the number of steps taken by a range of 2000–2500 per day [12].

Engagement with the app for Events indicates its value in patients monitoring their symptoms in real-time. The high number of reports of falls/near falls and choking/coughing supports natural history data since these are both disease symptoms known to be associated with disease progression. The limited number of tremor-related events may reflect the fact that tremor’s were also reported as part of the weekly mPROs and that this is not a consistent symptom in all patients.

With a small number of patients and a large number of variables, the correlation analysis aims to suggest relationships, rather than provide clear evidence. Figure 3 shows that highly active patients, as measured with the wearable, perform better at the clinical walking tests, and report lower disease impact. As the clinical assessments do not all seem to agree (i.e. ataxia does not show a negative relationship to walking performance), the combination of mPROs and wearable could provide additional information on disease impact. Increased correlation was seen between the wearable metrics and impact scales at month 6 compared to month zero. This was unexpected given that LOTS is a stable disease and there weren’t many changes in ADM, ADS or ADE over the 6-month study period. As this was a natural history study with a small sample size, it is not possible to rationalise these observations as the statistics are only indicative.

One of the insights gained through this study was that clinical measures do not always match patient self-perceived disease impact. For example, the patient with the highest reported score of Wider Impact and Tremor mPROs (008) had the third *least* disease impact according to the BARS score. However, the same patient reported the highest number of Events, and the highest number of healthcare visits (“Psychiatrist for physical therapy”, “MRI as part of natural history study”, “Phlebotomist”, “Speech Therapist”, “Neurologist”, “Psychologist”, “Dietician” and “Urologist”). This shows that the self-perceived impact of the disease is an important measure to consider in disease burden and may not correlate with clinical testing. The low perceived self-esteem of patients observed through their responses to the PROs, is expected in this patient population. Low self-esteem, emotional health and psychological issues are highly reported in patients with rare genetic disorders [Rare Disease UK 2018 – Living with a rare condition: the effect on mental health].

As a consequence of the feedback from the patient survey, many improvements have been made, and a new wearable device has been identified which will be integrated into future studies. Additional features will be developed including an integration of video conferencing and secure messaging to enable telemedicine consultations.

## Conclusions

In a highly motivated cohort of patients with a rare disease, mHealth and wearable technology was shown to be useful and feasible for capturing remote, real-time insight into disease burden. It is likely that a longer observation period will yield a clearer understanding of the nuances of disease progression and the individualized impact of disease burden that can be used as outcomes to therapeutic interventions.

## Methods

Patients were recruited at the National Institutes of Health in the USA, as part of an ongoing natural history study (02-HG-0107). All patients who were approached about the study consented to take part. Consenting patients were admitted for a three day stay for clinical assessments at baseline and at the 6-month completion of the trial including the Brief Ataxia Rating Scale (BARS) and subtest, the 6 min walk test (6MWT), neuroglyphics (a digital Archimedes spiral-drawing accuracy rating tool), the 9-hole peg test and GAITRite walking assessment.

**Fig. 4:**
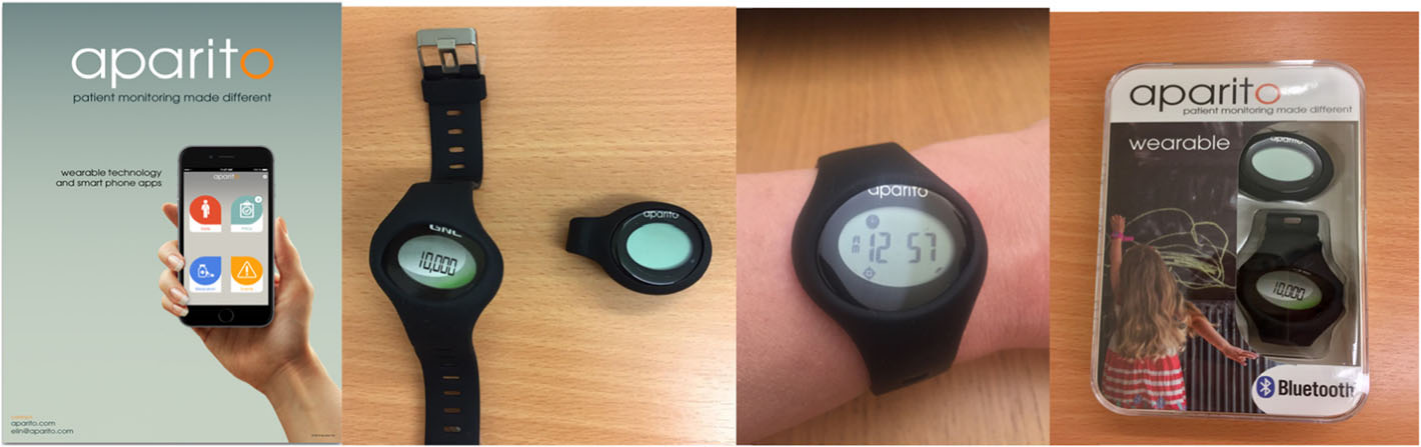
A Million Bluetooth pedometer wearable device was paired with an Aparito app (available on iOS and Android).

All consenting patients downloaded the Aparito app via Google or the App store (Android and iOS respectively) at the baseline visit and this was paired with a 3D accelerometer device to be worn on the wrist. Patients were asked to wear the 3D accelerometer continuously for the six-month duration of the study. The 3D accelerometer wrist-worn device captured data in 30-min epochs and calculated the number of steps taken for that 30-min period. The term ‘activity’ means patient engagement when wearing the device; activity does not mean physical activity in the context of this study (Fig. 4).

Three wearable metrics were computed as defined below:

i) The average daily maximum (ADM) is the maximum number of steps per epoch on each active day, averaged over all active days in the month.
ii) Average daily steps (ADS) is the total number of steps taken by a patient on active days in a month divided by the number of active days.
iii) The average daily steps per epoch (ADE) is calculated as follows. The total number of steps in an active day is divided by the number of active epochs. This is then further averaged over the number of active days in the month.

Patients with very low-level levels of activity in a month had their data for any month excluded from analysis if the number of active days in that month was less than six days.

The patient-facing app captured disease symptoms which patients could access to report any symptom or health-related problem in real-time on the app. The pre-configured health symptoms were already listed in the app as a drop-down menu: Choking / Coughing, Fall / Near Fall, Missed College / Work, Tremor, Other Illness, Other (Note: patients entered their symptoms/problem via free text for this category).

Ten mPROs were pushed to the app at pre-set intervals ranging from 8 to 60 days. The mPROS were the Tremor Impact Scale, Disease Impact Scale, Family Impact Scale, Wider Impact Scale, Impact Composite Scale, Perceived Stress, Global Self-worth, Rosenberg Self Esteem, CHU9D and PedsQL Multi-dimensional Fatigue scale. These are described in the Appendix. The different PROs were pushed out at varying schedules as described in Table 13 in the Appendix. It should be noted that four patients carried on using the App after the agreed 6-month study period, but these data are not reported in this paper.

Patients also had the ability to record health care appointments in a ‘Visits’ section, allowing patients to record planned or emergency visits to different health care professionals via the app provided. The pre-configured visits already listed in the app included general practitioner, healthcare professional and hospital. In addition to this, patients had the option to provide further detail of the visit.

All wearable, clinical and mPRO data were tested for overall trends between baseline and month six. The methods used were the Wilcoxon matched pairs test and the Hollander test for bivariate symmetry [13]. These tests take account of the nonparametric nature of some of the data and the presence of tied data.

Correlation testing was pre-planned before the start of the study. No adjustments of *p*-values for multiple comparisons was made due to the exploratory nature of the study. At both baseline and month six the relationships between wearable data and clinical and mPRO data and within the set of three wearables were tested using the Spearman’s rank correlation test. This approach tests between-patient correlation at one time point. Correlations with coefficient ≥ |0.6| were considered as moderate to strong relationships [14].

The rationale for testing all mPRO data against the three wearable metrics was to explore new PROs against the metrics because there are no disease-specific PROs currently available for LOTs. Therefore, the correlation analyses were exploratory.

Adherence for the device was calculated when a minimum of 4 h of data (i.e. 8 × 30-min epochs) were captured for that day. Adherence was calculated as the total number of days active on the device divided by the total number of days in the 6-month study period. Adherence for the PRO responses presented in Table 2 was calculated by dividing the total number of actual responses per month by the number of expected responses per month for all PRO surveys over the 6-month period multiplied by 100. The average adherence rate for each month was calculated by dividing the total number of actual responses by the number of patients (i.e. 8 patients) multiplied by 100.

To learn from the experience and to improve on the technical capabilities of the wearable device, patients were asked to answer a questionnaire at the end of the study. The questionnaire comprised five questions.

## Data Availability

The datasets used and/or analysed during the current study are available from the corresponding author on reasonable request.

## Appendix

### Tremor Impact Scale

This scale, developed specifically for this study as it is a known complication of disease, was available for patients to record tremor in a particular week, and its impact on their ability to perform tasks. This PRO has a scale from 0 to 3 for a single domain. The highest total score of 3 ‘Yes, I had a severe tremor that impeded my ability to perform everyday tasks.’ is interpreted as the highest severity of tremors and 0 reflected ‘No, I did not have a tremor”. The complete range of questions with their possible answers and corresponding quantitative values are shown in Table 5 in Appendix.

**Table 5:** Tremor

Tremor		
Question	Possible Answer	Numerical value
Did you experience a tremor this week?	No, I did not have a tremor.	0
	Yes, I had a mild tremor, but it did not interfere with my ability to perform everyday tasks.	1
	Yes, I had a moderate tremor that interfered with my ability to perform everyday tasks.	2
	Yes, I had a severe tremor that impeded my ability to perform everyday tasks.	3
**Total Score**		3

### Impact of Late Onset GM2 Scale

This scale was modified from the Niemann Pick -C Patient/Parent Reported Scale that was developed by the International Niemann-Pick Disease Association for use in their disease registry as an indicator for the impact of disease. The questions asked were the impact of their disease on the ability to walk, coordination, speech, ability to swallow and cognitive abilities. This PRO has a total of 5 domains, with a scale scoring range for each individual domain of 0–3 to 0–5 depending on the domain (Table 6 in Appendix). This PRO also has a total score range from 0 to 19, where a high score implies high disease impact on an individual.

**Table 6:** Impact of Late Onset GM2

Impact of Late Onset GM2			
Question	Possible Answer	Numerical Value	Numerical Value Converted (For composite scale)
This section relates to your ability to walk about from place to place. Please select the option that best describes how you are generally.	I have no problems walking about	0	0
	I am clumsy	1	0.2
	I am unsteady on my feet	2	0.4
	I can walk but only with assistance, particularly outdoors	3	0.6
	I normally use a wheelchair outdoors. Inside/at home I move about on my feet but need assistance	4	0.8
	I cannot walk at all and to get around I always use a wheelchair	5	1
This section relates to your ability to coordinate your movements. Please select the option that best describes how you are generally.	I have no problems coordinating movement	0	0
	My movements can be shaky or trembling	1	0.25
	I have some problems with coordinating movement	2	0.5
	I have problems with coordinating movement, but can still feed myself	3	0.75
	I cannot perform any activities independently and need help to do everything	4	1
This section relates to your speech. Please select the option that best describes how you are generally.	I have no problems with speech	0	0
	My speech can be difficult to understand	1	0.333
	My speech is very difficult to understand and really only people who know me very well can understand what I am saying	2	0.667
	I cannot speak, but can communicate in other ways	3	1
This section relates to your ability to swallow. Please select the option that best describes how you are generally.	I have no difficulty in swallowing	0	0
	I chew abnormally	1	0.25
	I sometimes have difficulty in swallowing, and may occasionally cough while eating or drinking	2	0.5
	I have difficulty in swallowing every day, frequently coughing or choking on food or drinks	3	0.75
	My difficulties in swallowing are so severe that I now must be fed by nasogastric tube or gastrostomy.	4	1
Do you think your cognitive abilities are impacted by late onset GM2 gangliosidosis? i.e. learning new skills, making decisions, following instructions, focusing your attention	Not at all	0	0
	A little bit	1	0.333
	Quite a bit	2	0.667
	Very much	3	1
**Total Score**		19	

### Impact on the family of a late onset GM2 Patient Scale

This scale was developed by the International Niemann-Pick Disease Association for use in their disease registry. This PRO allows patients to record how their disease impacted their family. Questions included whether family members had to give up things due to the patient’s illness (Table 7 in Appendix). This PRO has a total of 4 domains, with an individual domain scoring range of 0–3 for one domain, while the other domains have a negative scoring from 3 to 0, as shown in Table 7 in Appendix. This PRO has a total score range from 0 to 12, where a higher total score implies a higher negative impact on family life.

**Table 7:** Impact on the family of a late onset GM2 patient

Impact on the family of a late onset GM2 patient			
Question	Possible Answer	Numerical value	Converted Value (For composite scale)
I don’t have much time left over for other family members after caring for myself	Strongly Agree	3	1
	Agree	2	0.667
	Disagree	1	0.333
	Strongly Disagree	0	0
Our family gives up things because of my illness	Strongly Agree	3	1
	Agree	2	0.667
	Disagree	1	0.333
	Strongly Disagree	0	0
I worry about what will happen in the future	Strongly Agree	3	1
	Agree	2	0.667
	Disagree	1	0.333
	Strongly Disagree	0	0
Because of what we have shared we are a closer family	Strongly Agree	0	0
	Agree	1	0.333
	Disagree	2	0.667
	Strongly Disagree	3	1
**Total Score**		12	

### Wider Impact scale

This scale was also developed by the International Niemann-Pick Disease Association for use in their disease registry. This PRO allows patients to record the wider impact of late onset GM2. The questions asked include the impact on the jobs/school of family members, and visits to emergency rooms due to the illness. A full list of the questions and possible answers can be found in Table 8 in Appendix. This PRO has a total of 4 domains, with a domain scoring range of 0–3 and 0–4 which is dependent on the domain being considered. This PRO has a total score range from 0 to 14 where a higher score implies a wider negative impact of the disease.

**Table 8:** Wider impact of late onset GM2 gangliosidosis

Wider impact of late onset GM2 gangliosidosis			
Question	Possible Answer	Numerical value	Converted Numerical Value (For composite scale)
Over the past month, did you miss any time from your job or school due to your illness?	No	0	0
	Yes, 1 day	1	0.25
	Yes, 2 days	2	0.5
	Yes, 3 or 4 days	3	0.75
	I have been unable to work/study due to my illness or have changed my working pattern to be able to accommodate my illness	4	1
Over the past month, have other family members missed time from their job or school (i.e. college or university) due to your illness?	No	0	0
	Yes, 1 day	1	0.25
	Yes, 2 days	2	0.5
	Yes, 3 or 4 days	3	0.75
	Another family member has changed his/her working pattern to be able to care for me	4	1
How many times, during the past month, did you visit the emergency room because of issues relating to your illness?	Never	0	0
	1 time	1	0.33
	2–3 times	2	0.66
	More than 4 times	3	1
During the past month, how many appointments have you had with a physician regarding your illness?	None	0	0
	1 appointment	1	0.33
	2–3 appointments	2	0.66
	More than 4 appointments	3	1
**Total Score**		**14**	

### Impact Composite Scale

Impact Composite Scale consists of 3 different impact scales: the Impact of Late onset GM2 (Table 6 in Appendix), the Impact on the family of a late onset GM2 patient (Table 7 in Appendix), and the Wider Impact (Table 8 in Appendix). These were combined using a mean composite scoring system [15] to have a total score range from 0 to 1 where a higher score implies higher negative impact.

### Perceived Stress

This scale was taken from the Perceived Stress Scale, a psychological instrument used for measuring the perception of stress [16]. For this PRO, patients rate specific situations on how stressful they felt. This PRO has a total of 10 domains, with an individual domain scoring range of 0–4, and which also includes negative scoring from 4 to 0. The total scoring range from all domains is 0–40, where a high score implies a high amount of perceived stress. A full list of the questions and possible answers can be found in Table 9 in Appendix.

**Table 9:** Perceived Stress

Perceived Stress		
Question	Possible Answer	Numerical value
In the last month, how often have you been upset because of something that happened unexpectedly?	Never	0
	Almost Never	1
	Sometimes	2
	Fairly Often	3
	Very Often	4
In the last month, how often have you felt that you were unable to control the important things in your life?	Never	0
	Almost Never	1
	Sometimes	2
	Fairly Often	3
	Very Often	4
In the last month, how often have you felt nervous and “stressed”?	Never	0
	Almost Never	1
	Sometimes	2
	Fairly Often	3
	Very Often	4
In the last month, how often have you felt confident about your ability to handle your personal problems?	Never	4
	Almost Never	3
	Sometimes	2
	Fairly Often	1
	Very Often	0
In the last month, how often have you felt that things were going your way?	Never	4
	Almost Never	3
	Sometimes	2
	Fairly Often	1
	Very Often	0
In the last month, how often have you found that you could not cope with all the things that you had to do?	Never	0
	Almost Never	1
	Sometimes	2
	Fairly Often	3
	Very Often	4
In the last month, how often have you been able to control irritations in your life?	Never	4
	Almost Never	3
	Sometimes	2
	Fairly Often	1
	Very Often	0
In the last month, how often have you felt that you were on top of things?	Never	4
	Almost Never	3
	Sometimes	2
	Fairly Often	1
	Very Often	0
In the last month, how often have you been angered because of things that were outside of your control?	Never	0
	Almost Never	1
	Sometimes	2
	Fairly Often	3
	Very Often	4
In the last month, how often have you felt difficulties were piling up so high that you could not overcome them?	Never	0
	Almost Never	1
	Sometimes	2
	Fairly Often	3
	Very Often	4
**Total Score**		40

### Global Self Worth

This PRO scale was modified from the Global Self-Worth Scale [17], and was developed for patients to rate specific situations stated in the PRO on their self-worth. This PRO has a total of 6 domains, with an individual domain scoring range of 0–4 and which also includes negative scoring from 4 to 0 (Table 10 in Appendix). The total scale scoring range from all domains is 0–24, where a higher score implies a higher self-worth.

**Table 10:** Global Self Worth

Global Self Worth		
Question	Possible Answer	Numerical Value
Some people are often unhappy with themselves. BUT Other people are pretty pleased with themselves.	‘Some people are often unhappy with themselves.’ is really true for me.	1
	‘Some people are often unhappy with themselves.’ is sort of true for me.	2
	‘Other people are pretty pleased with themselves.’ is sort of true for me	3
	‘Other people are pretty pleased with themselves.’ is really true for me.	4
Some people don’t like the way they are leading their life. BUT Other people do like the way they are leading their life.	‘Some people don’t like the way they are leading their life.’ is really true for me.	1
	‘Some people don’t like the way they are leading their life.’ is sort of true for me.	2
	‘Other people do like the way they are leading their life.’ is sort of true for me	3
	‘Other kids do like the way they are leading their life.’ is really true for me.	4
Some people are happy with themselves as a person. BUT Other people are often not happy with themselves.	‘Some people are happy with themselves as a person.’ is really true for me.	4
	‘Some people are happy with themselves as a person.’ is sort of true for me.	3
	‘Other people are often not happy with themselves.’ is sort of true for me.	2
	‘Other people are often not happy with themselves.’ is really true for me.	1
Some people like the kind of person they are. But Other people often wish they were someone else.	‘Some people like the kind of person they are.’ is really true for me.	4
	‘Some people like the kind of person they are.’ is sort of true for me.	3
	‘Other people often wish they were someone else.’ is sort of true for me.	2
	‘Other people often wish they were someone else.’ is really true for me.	1
Some people are very happy being the way they are but other people wish they were different.	‘Some people are very happy being the way they are.’ is really true for me.	4
	‘Some people are very happy being the way they are.’ is sort of true for me.	3
	‘Other people wish they were different.’ is sort of true for me.	2
	‘Other people wish they were different.’ is really true for me.	1
Some people are not very happy with the way they do a lot of things but other people think the way they do things is fine.	‘Some people are not very happy with they way they do a lot of things.’ is really true for me.	1
	‘Some people are not very happy with they way they do a lot of things.’ is sort of true for me.	2
	‘Other people think the way they do things is fine.’ is sort of true for me.	3
	‘Other people think the way they do things is fine.’ is really true for me.	4
**Total Score**		24

### Rosenberg Self-Esteem

This PRO scale was modified from the Global Self-Worth Scale [18], and was developed for patients to rate specific situations stated in the PRO on their self-esteem. This PRO has a total of 10 domains, with an individual domain scoring range of 0–3 and which also includes negative scoring from 3 to 0 (Table 11 in Appendix). The total scale scoring range from all domains is 0–30, where a higher score implies a higher self-esteem.

**Table 11:** Rosenberg Self Esteem

Rosenberg Self-esteem		
Question	Possible Answer	Numerical value
On the whole, I am satisfied with myself.	Strongly Agree	3
	Agree	2
	Disagree	1
	Strongly Disagree	0
At times, I think I am no good at all.	Strongly Disagree	0
	Disagree	1
	Agree	2
	Strongly Agree	3
I feel that I have a number of good qualities.	Strongly Agree	3
	Agree	2
	Disagree	1
	Strongly Disagree	0
I am able to do things as well as most other people.	Strongly Agree	3
	Agree	2
	Disagree	1
	Strongly Disagree	0
I feel I do not have much to be proud of.	Strongly Disagree	0
	Disagree	1
	Agree	2
	Strongly Agree	3
I certainly feel useless at times.	Strongly Disagree	0
	Disagree	1
	Agree	2
	Strongly Agree	3
I feel that I’m a person of worth, at least on an equal plane with others.	Strongly Agree	3
	Agree	2
	Disagree	1
	Strongly Disagree	0
I wish I could have more respect for myself.	Strongly Disagree	0
	Disagree	1
	Agree	2
	Strongly Agree	3
All in all, I am inclined to feel hat I am a failure.	Strongly Disagree	0
	Disagree	1
	Agree	2
	Strongly Agree	3
I take a Positive attitude to myself	Strongly Agree	3
	Agree	2
	Disagree	1
	Strongly Disagree	0
**Total Score**		30

### CHU 9D

This PRO was taken from the Child Health Utility 9D (CHU 9D) (UK weighted tariff) [19], and was developed as a measure of a patient’s health related quality of life. This PRO has a total of 9 domains with an individual domain scoring range of weightings ranging from 0 to 0.1079 when considering all domains collectively (Table 12 in Appendix). The total scale scoring range from all domains is 0.33 to 1 where a higher score implies good health [20].

**Table 12:** CHU 9D

CHU9D		
Question	Possible Answer	Score of coded value
Worried	I don’t feel worried today	0
	I feel a little bit worried today	0.0227
	I feel a bit worried today	0.0227
	I feel quite worried today	0.0227
	I feel very worried today	0.0227
Sad	I don’t feel sad today	0
	I feel a little bit sad today	0.042
	I feel a bit sad today	0.0445
	I feel quite sad today	0.0722
	I feel very sad today	0.0722
Pain	I don’t have any pain today	0
	I don’t have any pain today	0.0322
	I don’t have any pain today	0.0322
	I don’t have any pain today	0.1245
	I don’t have any pain today	0.1426
Tired	I don’t feel tired today	0
	I feel a little bit tired today	0.0479
	I feel a bit tired today	0.0479
	I feel quite tired today	0.0479
	I feel very tired today	0.0479
Annoyed	I don’t feel annoyed today	0
	I feel a little bit annoyed today	0.0313
	I feel a bit annoyed today	0.0313
	I feel quite annoyed today	0.0313
	I feel very annoyed today	0.0313
School Work/ Homework (such as reading, writing, doing lessons)	I have no problems with my schoolwork/homework today	0
	I have a few problems with my schoolwork/homework today	0.0487
	I have some problems with my schoolwork/homework today	0.0487
	I have many problems with my schoolwork/homework today	0.0656
	I can’t do my schoolwork/homework today	0.0656
Sleep	Last night I had no problems sleeping	0
	Last night I had a few problems sleeping	0.0212
	Last night I had some problems sleeping	0.0212
	Last night I had many problems sleeping	0.0506
	Last night I couldn’t sleep at all	0.0907
Daily routine (things like eating, having a bath/shower, getting dressed)	I have no problems with my daily routine today	0
	I have a few problems with my daily routine today	0.0371
	I have some problems with my daily routine today	0.0612
	I have many problems with my daily routine today	0.0699
	I can’t do my daily routine today	0.093
Able to join in activities (things like playing out with your friends, doing sports, joining in things)	I can join in with any activities today	0
	I can join in with most activities today	0.0368
	I can join in with some activities today	0.0368
	I can join in with a few activities today	0.0368
	I can join in with no activities today	0.1079

### PedsQL™ Multidimensional Fatigue Scale

This PRO taken from the PedsQL™ Multidimensional Fatigue Scale [21] was used to measure the fatigue of the patients. This fatigue scale is formed of 18 items comprising of the General Fatigue Scale (6 items), Sleep/Rest Fatigue Scale (6 items), and Cognitive Fatigue Scale (6 items) Each individual domain is scored from 0 to 100, where based on intrinsic calculations, the total scale scoring range is also 0–100, where a higher score implies less problems with fatigue (Good).

### Patient Reporting and Timing

**Table 13:** PRO reporting timescales

Patient Reported Outcomes and Quality of Life Scales	Timing Schedule (Days)
Tremor Impact Scale	8
Impact of Disease Scale	28
Impact on the family Scale	28
Wider impact Scale	28
Perceived Stress	60
Global Self Worth	60
Rosenberg Self Esteem	60
CHU9D	60
General Fatigue	31
Sleep/Rest Fatigue	31
Cognitive Fatigue	31

## Abbreviations

ADE: Average daily steps per epoch
ADM: Average daily maximum
ADS: Average daily steps
BARS: Brief Ataxia Rating Scale
CHU 9D: Child Health Utility 9D
mPROs: Mobile Patient Reported Outcomes
6MWT: 6-min walk test
SD: Sandhoff disease
TSD: Tay-Sachs disease
